# Cultural adaptation for Brazil of the Child Abuse and Neglect Reporting Self-Efficacy Questionnaire

**DOI:** 10.1590/0034-7167-2024-0220

**Published:** 2025-10-03

**Authors:** Vitória Carla Conceição Almeida-Leandro, Beatriz Juliana Conacci, Jeyce Kelly dos Santos Oliveira, Vânia Pinheiro Ramos, Moacyr Lobo da Costa, Roberta Alvarenga Reis, Edson Zangiacomi Martinez, Stefanie Witt, Julia Hannah Quitmann, Monika Bullinger, Jeniffer Anne Fraser, Claudia Benedita dos Santos

**Affiliations:** I Universidade de São Paulo. Ribeirão Preto, São Paulo, Brazil; II Universidade Federal de Pernambuco. Recife, Pernambuco, Brazil; III Universidade Federal do Rio Grande do Sul. Porto Alegre, Rio grande do Sul, Brazil; IV University of Applied Sciences. Hamburgo, Germany; V University Medical Center Hamburg-Eppendorf. Hamburgo, Germany; VI University of Sydney. Sydney, Australia.

**Keywords:** Child, Health Personnel, Self Efficacy, Mandatory Reporting, Child Abuse, Niño, Personal de Salud, Autoeficacia, Notificación Obligatoria, Maltrato a los Niños

## Abstract

**Objectives:**

to culturally adapt the Child Abuse and Neglect Reporting Self-Efficacy Questionnaire for Brazil.

**Methods:**

a methodological study of clinical application, involving 126 healthcare professionals, developed between 2022 and 2023. The instrument measures healthcare professionals’ self-efficacy in reporting cases of child abuse and neglect. The translation process into Brazilian Portuguese and back-translation followed international standards, and face validity was implemented using an international method.

**Results:**

the Brazilian version is composed of two dimensions and 35 items, and was well assessed by participants, with the majority considering it good or very good (88.1%), with easy-to-understand questions (73.8%), without any difficulty with the answer options (74.6%) and very important for the situation (85.7%).

**Conclusions:**

the translated version preserved the conceptual equivalence of items, presenting face validity, and can be considered culturally adapted to the context studied.

## RESUMEN

**Objetivos:**

adaptar culturalmente el *Child Abuse and Neglect Reporting Self-Efficacy Questionnaire* a Brasil.

**Métodos:**

estudio metodológico de aplicación clínica, involucrando a 126 profesionales de la salud, desarrollado entre 2022 y 2023. El instrumento mide la autoeficacia de los profesionales de la salud para denunciar casos de abuso y negligencia infantil. El proceso de traducción al portugués, hablado y escrito en Brasil, y la retrotraducción siguieron estándares internacionales y la validez aparente se implementó mediante un método internacional.

**Resultados:**

la versión brasileña está compuesta por dos dimensiones y 35 ítems, siendo bien evaluada por los participantes, siendo la mayoría considerándola buena o muy buena (88,1%), con preguntas fáciles de entender (73,8%), sin dificultad con las opciones de respuesta (74,6%) y muy importantes para la situación (85,7%).

**Conclusiones:**

la versión traducida conservó la equivalencia conceptual de los ítems, presenta validez aparente y puede considerarse adaptada culturalmente para el contexto estudiado.

## INTRODUCTION

The World Health Organization defines violence as “the intentional use of physical force or power, threatened or actual, against oneself, another person, or against a group or community, that either results in or has a high likelihood of resulting in injury, death, psychological harm, maldevelopment or deprivation”^(1)^. The different types of violence are physical abuse or mistreatment, emotional or psychological abuse or mistreatment, sexual abuse or mistreatment, and neglect or abandonment^(2-4)^.

According to the Ministry of Human Rights and Citizenship, between January and April 2023, there were 69.3 thousand complaints and 397 thousand human rights violations against children and adolescents in Brazil^(5)^. In 2001, the Ministry of Health, through Ordinance 1968 of October 25, 2001, made available a reporting form for violence against children and adolescents for use by healthcare professionals when victims of violence were treated by the Brazilian Health System (In Portuguese, *Sistema Único de Saúde* - SUS). However, in 2006, through the publication of Law 4,725 of March 15, 2006, mandatory reporting became mandatory in cases of violence against children and adolescents for the three levels of healthcare and private institutions in Brazil^(2,6,7)^.

However, in practice, cases of violence are still underreported in Brazil. A lack of knowledge of reporting forms, less than five years of professional service and the fear of legal involvement that may result from being the author of the report were some of the reasons highlighted in a study carried out in 2014^(6,8,9)^. The problem of underreporting of cases is a priority, and the role played by healthcare professionals is crucial for policies to address violent acts and the consequences caused by this practice^(10)^. Moreover, it contributes directly to health surveillance, since it is through the reporting carried out by these professionals that violence becomes epidemiologically visible, through the feeding of the Notifiable Diseases Information System (In Portuguese, *Sistema de Informação de Agravos de Notificação* - SINAN)^(11)^.

In relation to self-efficacy in reporting cases, although reporting a situation of violence is mandatory by law, healthcare professionals who report do not have any special protection measures. It has already been identified in literature that one of the greatest fears of those reporting cases, especially in the context of primary healthcare, is suffering retaliation by perpetrators of aggression and further aggravating victims’ condition, considering that most of these professionals live in the community where they work professionally and “need to follow the rules that the ‘parallel power’ imposes through fear and terror”^(12-17)^. The need for adequate training to identify situations of violence against children and adolescents is of great importance, considering that this issue is a great challenge because it involves biopsychosocial situations that are complex to perceive and manage^(18-21)^.

Studies indicate that dentists are in a privileged position to identify possible situations of violence against children and adolescents, due to their expanded vision during clinical examinations, and can identify the regions most affected in cases of aggression in the neck, skull, face and oral cavity regions^(22,23)^. These scientific findings are evidenced by the study by Melo *et al*.^(24)^, who point out that dentists have mastery of knowledge about injuries caused to minors due to mistreatment, in addition to recognizing the Statute of Children and Adolescents as an appropriate guideline that emphasizes the obligation and duty of professionals who identify the situation of violence to report the case to authorities^(24)^.

Galindo *et al.* ^(25)^ state that, for nurses to identify a victim of violence, a trained clinical eye is required, which is one of the difficulties that nurses face on a daily basis when acting in situations of violence. In addition, the authors also list other realities, such as not exposing children, adolescents and their families to their aggressors even more. The lack of education/training for the entire multidisciplinary team and excess work that predisposes to burnout syndrome are factors that contribute to cases of violence being camouflaged^(25)^.

In the context of public healthcare services, the gateway to the SUS is the Family Health Strategy, and this space has a privileged position to identify victims who suffer abuse. Therefore, it is of great importance that these professionals are guided on how to detect cases and how to follow up so that victims receive the necessary assistance^(15)^. However, the bonds established between users and professionals should be seen and treated as positive points for the perception, monitoring and intervention of cases of violence against children and adolescents^(26)^. According to Fiocruz^(27)^, any professional who identifies a victim of violence must report it to SINAN and that no case can be underreported, considering that this is a mandatory report and that its completion is a dimension of healthcare. Thus, healthcare professionals’ experiences are relevant for identifying cases, either through the identification of injuries caused to victims or through conversations with victims and their families^(27)^.

The Principle of the Best Interests of the Child (PBIC) is a tool commonly used in decision-making involving children and/or adolescents, especially in the legal field. Even though the participants in this study are higher-level healthcare professionals, this is an unusual topic in clinical practice. It is used worldwide by professionals who work with children and adolescents, and is applied in the justice system^(28)^.

The difficulty in reporting in private healthcare services is also evident. The results of a study carried out by Pires *et al*.^(28)^, whose participants were pediatricians from Rio Grande do Sul who worked in public and private networks, revealed that some participants in the private network did not make mandatory reporting due to limited access to information on the subject, emotional involvement, fear of legal involvement and lack of effective institutional support in the private sector.

Considering the above, the object of study is the Child Abuse and Neglect Reporting Self-Efficacy Questionnaire (CANRSE), which measures healthcare professionals’ self-efficacy in reporting cases of child abuse and neglect. CANRSE was developed by Lee *et al*. in 2012^(29)^, Sydney, Australia, with the aim of assessing nursing professionals’ self-efficacy for reporting suspected or known cases of child and adolescent abuse and neglect. CANRSE, in its original version, consists of 55 items and three dimensions: confidence to report suspected cases; confidence to report known cases, both with 20 items; and confidence that reporting can produce positive results for the child and family, with 15 items^(30,31)^. For CANRSE adaptation process, the following stages were followed: translation, synthesis of translations, carried out by two translators and a recording observer, back-translation, review committee, pre-test and submission of the entire process to the authors of the instrument^(32-34)^.

This study is aligned with the Sustainable Development Goal 16, which aims to “Promote peaceful and inclusive societies for sustainable development, provide access to justice for all and build effective, accountable and inclusive institutions at all levels”^(35)^.

## OBJECTIVES

To culturally adapt CANRSE to Brazil.

## METHODS

### Ethical aspects

This research is part of research project entitled “Self-efficacy for Reporting Child Abuse and Neglect”, approved by a Research Ethics Committee Involving Human Beings.

### Study design, period and place

This is a methodological study of clinical application^(36,37)^, developed between 2023 and 2024 in the five regions of Brazil.

### Population or sample; inclusion criteria

The population of this study consists of healthcare professionals with higher education and who work or have worked for at least one year in the care of children and adolescents in Brazil. To compose the sample, participants were distributed into 14 subsets, formed according to the categorization of healthcare professionals into two subsets, such as those with one year or less than four years of training and those with five or more years of training, with each of these subsets subdivided into seven groupings of CANRSE items, namely: 1 to 5; 6 to 10; 11 to 15; 16 to 20; 21 to 25; 26 to 30; 31 to 35.

For each subset formed, at least three participants were considered, with an estimated sample of 42 for each of the five regions of Brazil, resulting in an estimated value of 210 healthcare professionals.

### Translation and back-translation into Brazilian Portuguese

The process of translation and back-translation of CANRSE items followed that proposed by Guillemin *et al*.^(33)^ and the adaptation proposed by Ferrer *et al.* ^(34)^. Translation was performed by two Brazilian researchers with knowledge of the subject and fluent in English. The expert committee was composed of five Brazilian researchers with a minimum doctoral degree, experience in translation, validity of instruments, expertise in the subject in question, knowledge of the research objectives and fluency in English. Back-translation was performed by a sworn translation professional, duly registered with the Commercial Board of São Paulo (In Portuguese, *Junta Comercial do Estado de São Paulo*).

### Face validity of the translated version for Brazil

During this stage, the semantic assessment method proposed by the DISABKIDS group^(38)^ was used, adapted for Brazil by the National Council for Scientific and Technological Development Research Group on Health Measures (GPEMSA-CNPq)^(39)^. Study participants assessed CANRSE using the general impression sheet made available by DISABKIDS^(38)^, adapted for Brazil by GPEMSA-CNPq^(39)^, containing three subjective questions and four objective questions. There were also responses regarding the specific printout, in which participants of each group answered the three questions (Table 1). Regarding clarity and relevance or representativeness and relevance, responses were described, according to a Likert-type scale, in four options, with 1 being the worst judgment of the questionnaire, and 4, the best. Responses 3 or 4, respectively, were considered satisfactory or very satisfactory in relation to questionnaire clarity and relevance/representativeness.

**Table 1:** Distribution of participants in relation to the questions contained in the specific semantic validity sheet according to CANRSE-35 BR items and category of longest professional experience with children and young people, Brazil, 2023

Child Abuse and Neglect Reporting Self-Efficacy Questionnaire-35BRASIL items	*Isto é importante para a sua situação?*				*O (A) senhor (a) teve dificuldade para entender essa questão?*		*O (A) senhor (a) poderia me dizer, em suas palavras, o que essa questão significa?*
Subset 1 (n =18)		Yes	Sometimes	No	Yes	No	
1		15	1	0	3	14	
2		14	2	1	2	15	
3		14	2	0	4	12	
4		14	3	0	4	13	
5		15	2	0	5	12	
Subset 2 (n =14)							
6		12	1	0	3	10	
7		12	1	0	2	10	
8		12	0	0	7	5	
9		11	0	0	2	9	
10		10	0	0	3	7	
Subset 3 (n =10)							
11		8	0	0	1	8	I was unsure whether the question means that in cases of suspected physical abuse I can count on my coordinator’s opinion or whether I am convinced that it is my right to seek him out in this situation and count on his help.
12		9	0	0	3	6	1. If I have doubts about how to report cases of sexual abuse, I can ask my supervisor for her opinion. 2. I was unsure whether the question means that in cases of sexual abuse I can count on my coordinator’s opinion or whether I am convinced that it is my right to seek him out in this situation and count on his help.
13		7	1	0	2	7	
14		8	1	0	4	5	
15		9	0	0	4	5	
Subset 4 (n = 8)							
16		8	0	0	3	5	
17		8	0	0	2	6	
18		8	0	0	2	6	
19		7	1	0	2	6	
20		8	0	0	2	6	
Subset 5 (n =11)							
21		7	2	2	1	8	I could not understand the principle of the best interests of the child.
22		8	1	2	2	8	
23		7	2	2	2	7	
24		7	2	2	2	7	The principle of the best interests of the child is confusing.
25		8	0	3	2	7	The principle of the best interests of the child - confusing.
Subset 6 (n = 8)							
26		7	0	0	3	4	The issue is confusing, perhaps because the issue of the child’s best interests is a topic little discussed by healthcare professionals.
27		7	0	0	2	5	
28		6	0	0	2	4	
29		6	0	0	2	4	
30		6	0	0	2	4	
Subset 7 (n = 6)							
31		5	1	0	2	4	
32		5	1	0	0	6	
33		5	1	0	0	6	
34		5	1	0	0	6	
35		5	1	0	0	6	

### Procedure for data collection

**Figure 1:**
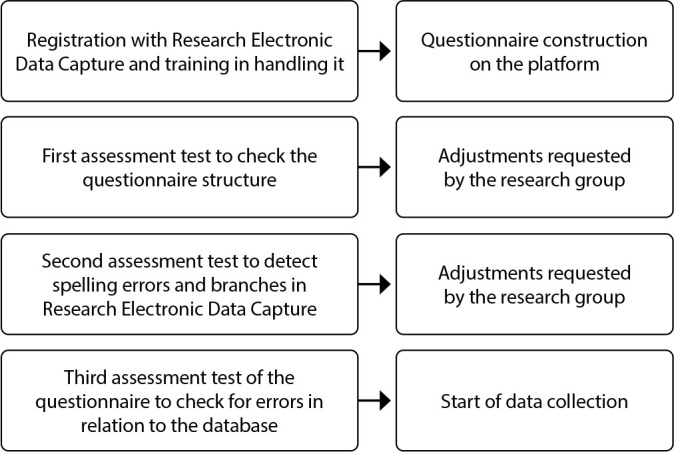
Flowchart of the stages for making data collection available through REDCap, Ribeirão Preto, São Paulo, Brazil, 2023

During this stage, to compose the judging committee, websites of the country’s higher education institutions were consulted, and professionals were selected using information contained in the *Lattes* Platform. Participants were selected from the Northeast, South and Southeast regions (São Paulo and Minas Gerais). Each participant received an invitation letter via email with information about the research and the date of the consensus meeting. To collect data for the semantic assessment stage, participants received an invitation letter via email and through open networks explaining the research. Consent to participate was obtained via an electronic form on the Research Electronic Data Capture (REDCap) platform, with the “yes” option selected after reading the Informed Consent Form (ICF). Only after this did participants have access to the instrument available on the platform. Each stage was assessed by participants from research GPEMSA group (Figure 1).

## RESULTS

**Figure 2:**
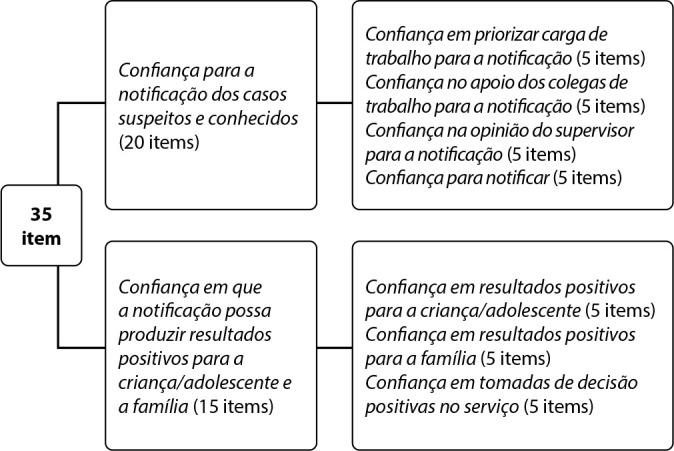
Theoretical Model to be confirmed according to factor analysis of the adapted version for Brazil of the Child Abuse and Neglect Reporting Self-Efficacy Questionnaire (CANRSE-35BR)

As for translation and back-translation, the researcher responsible for developing the instrument agreed with the content of each item, preserving the measured construct. However, she suggested replacing “report” with “notify” and unifying the “confidence to report suspected cases” and “confidence to report known cases” dimensions, making CANRSE, in the Brazilian version, to consist of two dimensions with 35 items. The version of CANRSE for Brazil was called CANRSE-35BR (Figure 2).

In total, the study had 217 accesses, from the five regions of Brazil, to the instrument made available on REDCap, but 75 (34.6%) completed the responses to the specific validity sheet. In the general assessment phase, 126 healthcare professionals answered the 35 questions of CANRSE-35BR, with the Northeast region having the largest number of participants: 78 (61.9%).

Concerning participants’ level of education, the majority had a graduate degree (101; 80.2%), and regarding professional training, the majority (67; 53.2%) were nursing professionals. Among the participants, 56 (44.4%) had between one and four years of experience working with children and young people; and 70 (55.6%) had more than five years of experience working with children and young people.

CANRSE-35BR was very well assessed by participants, considering the questionnaire good or very good (111; 88.1%), with easy-to-understand questions (93; 73.8%), with no difficulty with the answer options (94; 74.6%) and very important for the situation (108; 85.7%). Also, the majority (106; 84.1%) would not like to change anything; did not want to add anything (115; 91.3%) and wanted to answer all the questionnaire questions (123; 97.6%).

Regarding discursive questions, the results are presented below:

Item: “*O (A) senhor (a) gostaria de mudar alguma coisa no questionário?*”. Among the participants, (18; 14.3%) reported wanting to change something in the questionnaire and suggested a clarification about the “best interests of the child and family”; Item: “*O (A) senhor (a) gostaria de acrescentar alguma coisa no questionário?*”. A few suggestions for additions were made by ten (7.9%) of the participants, with the creation of a field so that professionals could express themselves more and the inclusion of questions:

O (A) senhor (a) tem certeza do acompanhamento psicossocial das famílias das crianças/adolescentes suspeitas ou confirmadas de violência infantil?

O (A) senhor (a) tem certeza que pode notificar casos suspeitos ou confirmados de violência infantil por meio do disque 100, mantendo seu nome em anonimato?

O (A) senhor (a) tem segurança em identificar os abusos e negligência?

Considering that identifying is as important as reporting child abuse and neglect, one participant suggested adding the questions:

O (A) senhor (a) tem vivências prévias de notificação?

Houve apreensão de conhecimento a respeito ainda na graduação?

The suggestions were discussed and the decision was made not to accept them, as they would alter the content of the instrument and were suggested by a minority of the participants.

The question aimed to identify whether professionals have been involved in this topic since their academic training, currently applying it as a trained professional. Item: “*Teve alguma questão que o (a) senhor (a) não quis responder?*”. In this item, there were two responses from participants who did not want to answer the questions. However, they did not specify which questions, nor the reason, and since the questions did not have mandatory answers, they continued the survey until semantic assessment.

Seventy-five (34.6%) professionals participated in the entire semantic assessment, following the DISABKIDS method adapted for Brazil^(39)^, and initially answered the ten sociodemographic questions. Afterwards, they answered 35 items from CANRSE-35BR, six questions from the general validity sheet and the entire specific validity sheet.

The distribution of participants in this phase, in relation to the highest level of education, included 17 (22.7%) with an undergraduate degree and 58 (77.3%) with a graduate degree (specialization, residency, master’s, doctoral degree and post-doctoral degree). In relation to gender, 55 (73.3%) declared themselves to be female; 14 (18.7%) declared themselves to be male; and six (8.0%) preferred not to declare their gender. Regarding the place of work, the majority (61; 81.3%) of participants work professionally in urban areas; four (5.3%) work in rural areas; nine (12.0%) work in both locations (rural and urban areas); and one (1.3%) participant did not inform their place of work.

Using a specific assessment sheet, participants assessed each CANRSE-35BR item regarding the importance of items for their reality as a healthcare professional (*Isto é importante para a sua situação?*) regarding the difficulty in understanding the item “*O (A) senhor (a) teve dificuldade para entender essa questão?*”. Participants were also given the opportunity to express, in their own words, what the item they were responding to meant (*O (A) senhor (a) poderia me dizer, em suas palavras, o que essa questão significa?*) (Table 1).

Table 1 results show that, for each item, the majority of respondents considered them “important to the situation”, with the minimum positive responses for this category being 14 (77.8%), 10 (71.4%), 7 (70.0%), 7 (87.5%), 7 (63.6%), 6 (75.0%) and 5 (83.3%) for subsets 1 to 7, respectively. Concerning doubts, these were associated with PBIC. Of the respondents to items 11 and 12, four participants expressed that they had doubts in understanding, as they did not know whether the question was about them knowing that they could count on the support of managers/supervisors to make the reporting in situations of abuse and/or neglect or whether, when situations like these arise, managers/supervisors work together, offering support and subsidies for reporting.

## DISCUSSION

The clarity and objectivity of a measuring instrument are essential factors for considering it good. Long texts and/or extensive sentences with a wealth of details can result in monotonous and exhaustive reading, causing respondents to become distracted and compromising their use^(40)^. Keszei *et al*.^(41)^ state that the use of ambiguous and/or vague terms can generate imprecise and inappropriate responses.

In this work, the unification of the dimensions “confidence to report suspected cases” and “confidence to report known cases” into “confidence to report suspected and known cases” reduced the initial number of 55 items by approximately 36%, making CANRSE, in the Brazilian version, an objective instrument, consisting of two dimensions, with 35 items.

In relation to relevance, clarity and comprehensibility, CANRSE-35BR was very well assessed by the 126 participants in this phase, with 111 (88.1%) considering it good or very good, with questions that were easy to understand (93; 73.8%), without any difficulty with the answer options (94; 74.6%) and very important for the situation (108; 85.7%). Furthermore, 115 (91.3%) did not want to add anything to the questionnaire, and 123 (97.6%) wanted to answer all the questions.

In relation to discursive questions, participants had the opportunity to express their opinions about CANRSE-35BR and to make suggestions and modifications to it. Once again, results were very satisfactory, with few suggestions made by participants, confirming the face validity of the instrument.

Regarding the essay question of the specific phase of semantic assessment, study participants had the opportunity to express the meaning of the item in their own words. The relevant result is associated with PBIC, a topic considered complex and difficult to understand by some participants.

Although 108 (85.7%) of participants assessed the others as relevant to their situation, the items that addressed PBIC were indicated as not important. The expression “The Best Interests of the Child” was used for the first time in 1924, in the Geneva Declaration of the Rights of the Child^(42)^. PBIC originally appeared in the text of the 1989 United Nations Convention on the Rights of the Child, when States’ obligations towards childhood were presented, determining the minimum that each nation should guarantee to its children and adolescents, in force from 1990 (UNICEF)^(43)^.

PBIC is important when children/adolescents are exposed to situations of mistreatment, neglect, or is going through the adoption, custody or foster care process. Actions that raise awareness among professionals about the effectiveness of reporting are essential, since reporting violence against children and adolescents is a first step towards controlling the problem^(44,45)^.

It is through semantic assessment, or face validity, that it is possible to make adjustments to the instrument to which it is intended to be adapted, enabling it to become as comprehensible as possible to the target population. During this stage, difficulties in understanding some terms may also be identified, as they are words that are not part of the target population’s experience^(46,47)^. The results presented showed that the translation process into Brazilian Portuguese and back-translation of CANRSE preserved conceptual equivalence of items with the adapted version, presenting face validity.

### Study limitations

In this study, although the invitation to participate was extended to all healthcare professionals, the majority of participants were nurses, with little participation from others. Furthermore, understanding that the topic on PBIC is more worked on in legal issues, this would be the justification for healthcare professionals not being familiar with the topic on “principle of the best interest”.

### Contributions to nursing, health or public policy

The results of this study revealed weaknesses related to the knowledge of PBIC by the participating healthcare professionals, which suggests the strengthening of public policies aimed at the training process and continuing education with a view to developing strategies aimed at effective reporting and monitoring of cases of violence against children and adolescents.

Also, from an interprofessional and multidisciplinary perspective, the provision of an instrument to measure the self-efficacy of reporting cases of child abuse and neglect can have a positive impact on several areas, such as clinical practice in nursing, enabling effective decision-making, supporting the management of cases of violence, the response of competent authorities and the safety of vulnerable children, adolescents and their families, as well as the professionals who report cases.

## CONCLUSIONS

This study concludes the process of cultural adaptation of CANRSE to the context studied, an instrument that, after being validated in relation to psychometric aspects, will constitute a valid and reliable tool that can be used to measure healthcare professionals’ self-efficacy to report suspected or known cases of child abuse and neglect, and to identify associated factors or to assess interventions for their improvement.

The Brazilian version of CANRSE, called CANRSE-35 BR, presented face validity and evidence of applicability, and can be considered culturally adapted for the country. Once again, the semantic assessment method, which provides an opportunity to “give voice” to study participants, allowed the objective of the process of cultural adaptation of instruments to be achieved with methodological rigor.

Although it is not an objective of this study, it is recommended that training be provided to detect and report suspected or known cases of violence against children and adolescents and other types of violence. To ensure better quality in the data analyzed, it is necessary to monitor reports, provide ongoing training for healthcare professionals, correctly identify suspected cases and fill out the reporting form appropriately. Actions that raise awareness among professionals regarding the effectiveness of reporting are essential, since reporting violence against children and adolescents is the first step towards controlling the problem^(47)^.

Thus, simultaneously with the verification of factor structure and validity of CANRSE, there is a need to develop studies that address PBIC in the context of healthcare service provision, since this study also clearly shows the lack of knowledge of some of the participants on this subject.

## Data Availability

The research data are not available.

## References

[1] World Health Organization (WHO) (2002). World report on violence and health.

[2] Governo do Estado do Rio de Janeiro (2006). Lei nº 4.725, de 15 de março de 2006. Cria a obrigação de notificação compulsória nos casos de violência contra criança e adolescentes.

[3] Krug EG, Dahlberg LL, Mercy JA, Zwi AB, Lozano R. (2002). World report on violence and health.

[4] Mathews B, Parcella R, Dunne M, Simunivic M, Marston C. (2020). Improving measurement of child abuse and neglect: a systematic review and analysis of national prevalence studies. PLoS ONE.

[5] Ministério da Saúde (BR), Secretaria de Vigilância em Saúde, Departamento de Vigilância Epidemiológica (2023). Sistema de informação de Agravos de Notificação - SINAN. Violência Interpessoal/Autoprovocada.

[6] Ministério da Saúde (BR) (2001). Dispõe sobre a notificação, às autoridades competentes, de casos de suspeita ou de confirmação de maus-tratos contra crianças e adolescentes atendidos nas entidades do Sistema Único de Saúde.

[7] Ministério da Saúde (BR) (2023). VIVA/SINAN - Vigilância Contínua.

[8] Presidência da República (BR) (1990). Dispõe sobre o Estatuto da Criança e do Adolescente e dá outras providências.

[9] Fórum Brasileiro de Segurança Pública (FBSP) (2023). Anuário Brasileiro de Segurança Pública 2022: Violência sexual infantil, os dados estão aqui, para quem quiser ver.

[10] Cecilia M, Deslandes SF. (2003). Caminhos do pensamento: epistemologia e método.

[11] Ministério da Saúde (BR) (2016). Viva: instrutivo notificação de violência interpessoal e autoprovocada.

[12] Martins AKRS, Rezaghi CJ, Nunes R. (2024). A importância da notificação de violência interpessoal e/ou autoprovocada no SINAN no âmbito da atenção básica: relato de experiência. Health Resid J (HRJ).

[13] Silva BP, Camargo D. (2023). As práticas profissionais realizadas em situações de maus-tratos infantis: uma revisão integrativa. Ciên Saúde Coletiva.

[14] Ministério da Saúde (MS) (2011). Portaria nº 104, de 25 de janeiro de 2011. Define as terminologias adotadas em legislação nacional, conforme o disposto no Regulamento Sanitário Internacional 2005 (RSI 2005), a relação de doenças, agravos e eventos em saúde pública de notificação compulsória em todo o território nacional e estabelece fluxo, critérios, responsabilidades e atribuições aos profissionais e serviços de saúde.

[15] Freitas RJM, Lima CLF, Costa TAM, Barros AS, Moura NA, Monteiro ARM. (2021). Violência intrafamiliar contra criança e adolescente: o papel da enfermagem. Rev Pesq Cuid Fundam.

[16] Leite JCS, Albuquerque GA. (2023). A Estratégia Saúde da Família e o enfrentamento à violência contra crianças e adolescentes: revisão integrativa. Ciênc Saúde Coletiva.

[17] Marques VJRC, Silva ABRC, Ayres AMN, Pagels CR, Toni LP, Silva CCS. (2021). Atenção primária à saúde e apoio psicossocial a crianças e adolescentes vítimas de violência doméstica: revisão integrativa. Rev Ciênc Saúde Nova Esperança.

[18] Ferreira VCBC, Costa JKA, Ferreira ALM, Ferreira BO, Torres MS. (2023). Punição corporal em crianças e adolescentes: uma revisão de escopo. Rev Interfaces Saúde Humanas Tecnol.

[19] Muniz BAA, Dantas ALM, Santana MM. (2022). Notificação de violência infantojuvenil: percepção dos profissionais da Atenção Primária à Saúde. Trab Educ Saúde.

[20] Maia MA, Silva MAC, Paiva ACO, Silva DM, Alves M. (2020). Práticas profissionais em situações de violência na atenção domiciliar: revisão integrativa. Ciênc Saúde Coletiva.

[21] Freitas RJM, Moura NA, Bessa MM, Lima LS, Monteiro ARM. (2022). Violência contra crianças e adolescentes em sofrimento psíquico: percepção dos profissionais de saúde. Saúde Redes.

[22] Matos ÉMR, Silva HKC, Nascimento FS. (2020). A responsabilidade civil e legal do cirurgião dentista frente a crianças e adolescentes que sofrem maus-tratos. Rev Cathedral.

[23] Melo JGA, Araújo LNC, Soares AML, Soares DM. (2023). Conhecimento dos cirurgiões-dentistas brasileiros na detecção de maus-tratos infantis. Arch Health Invest.

[24] Galindo NAL, Gonçalves CFG, Galindo NM (2023). Child and youth violence under the perspective of nursing. Rev Enferm UFPE.

[25] Nunes MCA, Morais NA. (2021). Práticas profissionais relacionadas às demandas de violência sexual: revisão da literatura nacional. Psicol Ciênc Prof.

[26] Farias E. (2023). Especialistas abordam importância da notificação de casos de violência contra crianças e jovens.

[27] Pires JM, Goldani MZ, Vieira EM, Nava TR, Feldens L, Castilhos K (2005). Barreiras para a notificação pelo pediatra de maus-tratos infantis. Rev Bras Saúde Mater Infant.

[28] Florenzano BP. (2021). Princípio do melhor interesse da criança: como definir a guarda dos filhos?.

[29] Lee PY, Dunne MP, Chou FH, Fraser JA. (2012). Development of the child abuse and neglect reporting self-efficacy questionnaire for nurses. Kaohsiung J Med Sci.

[30] Colgrave J, Stasa H, Fraser J. (2020). Validity and reliability of the psychometric properties of a child abuse questionnaire. Nurse Res.

[31] Colgrave J. (2021). Child abuse and neglect reporting: professional self-efficacy in Australian regional and rural emergency departments.

[32] Beaton DE, Bombardier C, Guillemin F, Ferraz MB. (2000). Guidelines for the process of cross-cultural adaptation of self-report measures. Spine.

[33] Guillemin F, Bombardier C, Beaton D. (1993). Cross-cultural adaptation of health-related quality of life measures: literature review and proposed guidelines. J Clin Epidemiol.

[34] Ferrer M, Alonso J, Prieto L, Plaza V, Monsó E, Marrades R (1996). Validity and reliability of the St George’s Respiratory Questionnaire after adaptation to a different language and culture: the Spanish example. Eur Respir J.

[35] Organização das Nações Unidas (ONU) (2023). Sobre o nosso trabalho para alcançar os Objetivos de Desenvolvimento Sustentável no Brasil.

[36] Lobiondo-Wood G, Haber J, Titler MG. (2019). St.

[37] Polit DF, Beck CT. (2018). Fundamentos de pesquisa em enfermagem.

[38] Romeiro V, Bullinger M, Marziale MHP, Fegadolli C, Reis RA, Silveira RCCP (2020). DISABKIDS® in Brazil: advances and future perspectives for the production of scientific knowledge. Rev Latino-Am Enfermagem.

[39] Dantas RAS, Silva FS, Ciol MA. (2013). Psychometric properties of the Brazilian Portuguese versions of the 29- and 13-item scale of the Antonovsky’s Sense of Coherence (SOC-29 and SOC-13) evaluated in Brazilian cardiac patients. J Clin Nurs.

[40] Mokkink LB, Terwee CB, Patrick DL, Alonso J, Stratford PW, Knol DL (2010). The COSMIN checklist for assessing the methodological quality of studies on measurement properties of health status measurement instruments: an international Delphi study. Qual Life Res.

[41] Keszei AP, Novak M, Streiner DL. (2010). Introduction to health measurement scales. J Psychosom Res.

[42] Custer LB. (1978). The origins of the doctrine of parens patriae. Emory LJ.

[43] UNICEF Brasil (1990). Convenção sobre os direitos da criança.

[44] Mendes JAA, Ormerod T. (2019). O princípio dos melhores interesses da criança: uma revisão integrativa de literatura em inglês e português. Psicol Estud.

[45] Oliveira NF, Moraes CL, Junger WL, Reichenheim ME. (2020). Violência contra crianças e adolescentes em Manaus, Amazonas: estudo descritivo dos casos e análise da completude das fichas de notificação, 2009-2016. Epidemiol Serv Saúde.

[46] Lima ACMACC, Bezerra KC, Sousa DMN, Rocha JF, Oriá MOB. (2017). Development and validation of a booklet for prevention of vertical HIV transmission. Acta Paul Enferm.

[47] Pasquali L. (2010). Instrumentação psicológica: fundamentos e práticas.

