# Supplementary material for: Cultural adaptation for Brazil of the Child Abuse and Neglect Reporting Self-Efficacy Questionnaire
Source: Rev Bras Enferm. 2025 Oct 3;78(4):e20240220. doi: 10.1590/0034-7167-2024-0220 (PMC12494354; doi:10.1590/0034-7167-2024-0220)
Supplement: Translation

# Translation: Adaptação cultural para o Brasil do *Child Abuse and Neglect Reporting Self-Efficacy Questionnaire*

**DOI:** 10.1590/0034-7167-2024-0220pt

**Authors:** Vitória Carla Conceição Almeida-Leandro, Beatriz Juliana Conacci, Jeyce Kelly dos Santos Oliveira, Vânia Pinheiro Ramos, Moacyr Lobo da Costa, Roberta Alvarenga Reis, Edson Zangiacomi Martinez, Stefanie Witt, Julia Hannah Quitmann, Monika Bullinger, Jeniffer Anne Fraser, Claudia Benedita dos Santos

**Affiliations:** I Universidade de São Paulo. Ribeirão Preto, São Paulo, Brasil; II Universidade Federal de Pernambuco. Recife, Pernambuco, Brasil; III Universidade Federal do Rio Grande do Sul. Porto Alegre, Rio grande do Sul, Brasil; IV University of Applied Sciences. Hamburgo, Alemanha; V University Medical Center Hamburg-Eppendorf. Hamburgo, Alemanha; VI University of Sydney. Sydney, Austrália.

**Keywords:** Criança, Pessoal da Saúde, Autoeficácia, Notificação de Abuso, Maus-Tratos Infantis

## Abstract

**Objetivos:**

adaptar culturalmente o *Child Abuse and Neglect Reporting Self-Efficacy Questionnaire* para o Brasil.

**Métodos:**

estudo metodológico de aplicação clínica, envolvendo 126 profissionais de saúde, desenvolvido entre 2022 e 2023. O instrumento mensura a autoeficácia dos profissionais de saúde para a notificação de casos de abuso e negligência infantil. O processo de tradução para a língua portuguesa, falada e escrita no Brasil, e retrotradução seguiu padrões internacionais, e a validade de face foi implementada usando método internacional.

**Resultados:**

a versão brasileira está composta por duas dimensões e 35 itens, sendo bem avaliada pelos participantes, com a maioria o considerando bom ou muito bom (88,1%), com questões fáceis de entender (73,8%), sem qualquer dificuldade com as opções das respostas (74,6%) e muito importantes para a situação (85,7%).

**Conclusões:**

a versão traduzida preservou equivalência conceitual dos itens, apresentando validade de face, e pode ser considerada adaptada culturalmente para o contexto estudado.

## INTRODUÇÃO

A Organização Mundial da Saúde define violência como “o uso de força física ou de poder, em forma de ameaça ou praticada, contra si próprio, contra outra pessoa ou contra um grupo ou comunidade que resulte ou que possa resultar em sofrimento, morte, desenvolvimento físico, mental ou social prejudicado ou privação”^(1)^. Os diferentes tipos de violência são abuso ou maus-tratos físico, abuso ou maus-tratos emocional ou psicológico, abuso ou maus-tratos sexual e negligência ou abandono^(2-4)^.

Segundo o Ministério dos Direitos Humanos e da Cidadania, entre janeiro e abril de 2023, ocorreram 69,3 mil denúncias e 397 mil violações de direitos humanos contra crianças e adolescentes no Brasil^(5)^. Em 2001, o Ministério da Saúde, por meio da Portaria nº 1968, de 25 de outubro de 2001, disponibilizou uma ficha de notificação de violência contra crianças e adolescentes para utilização pelos profissionais de saúde quando as vítimas de violência fossem atendidas no Sistema Único de Saúde (SUS). Todavia, em 2006, mediante a publicação da Lei nº 4.725, de 15 de março de 2006, houve a obrigatoriedade de notificação compulsória nos casos de violência contra criança e adolescente para os três níveis de atenção à saúde e instituições privadas do Brasil^(2,6,7)^.

Mas, ainda assim, na prática, os casos de violência são ainda subnotificados no Brasil. A falta de conhecimento da ficha de notificação, o tempo de serviço profissional inferior a cinco anos e o receio do envolvimento legal que pode resultar por ser o autor da notificação foram alguns dos motivos evidenciados em estudo realizado em 2014^(6,8,9)^. O problema da subnotificação dos casos é prioritário, e o papel desempenhado pelos profissionais de saúde é crucial para as políticas de enfrentamento dos atos violentos e das consequências ocasionadas por esta prática^(10)^. Além disso, contribui diretamente para a vigilância em saúde, pois é por intermédio da notificação que esses profissionais executam que a violência se torna visível epidemiologicamente, por meio da alimentação do Sistema de Informação de Agravos de Notificação (SINAN)^(11)^.

Em relação à autoeficácia para a notificação dos casos, embora o ato de notificar situação de violência seja uma obrigatoriedade instituída por lei, o profissional de saúde que notifica não dispõe de qualquer medida de proteção especial. Já é identificado na literatura que um dos maiores receios dos notificantes, principalmente no contexto da Atenção Primária à Saúde, é sofrer retaliações pelo autor da agressão e agravar ainda mais o quadro da vítima, considerando que a maioria desses profissionais moram na comunidade que atuam profissionalmente e “precisam seguir as regras que o ‘poder paralelo’ impõe por meio do medo e terror”^(12-17)^. A necessidade de capacitações adequadas para identificar situações de violência contra crianças e adolescentes é de grande importância, considerando que esta temática é um grande desafio por envolver situações biopsicossociais de complexa percepção e condução^(18-21)^.

Estudos indicam que os odontólogos têm posição privilegiada para identificação das possíveis situações de violência ao público infantojuvenil, devido à visão ampliada durante o exame clínico, podendo identificar as regiões mais acometidas em casos de agressões nas regiões de pescoço, crânio, face e cavidade oral^(22,23)^. Esses achados científicos são evidenciados por meio do estudo de Melo *et al.* ^(24)^, que apontam os cirurgiões-dentistas com domínio do conhecimento sobre as lesões ocasionadas a menores devido a maus-tratos, além de reconhecerem o Estatuto da Criança e do Adolescente como diretriz apropriada que enfatiza a obrigação e o dever do profissional que identifica a situação de violência realizar a notificação do caso às autoridades^(24)^.

Galindo *et al.* ^(25)^ afirmam que, para que os enfermeiros realizem a identificação de que há uma vítima de violência, exige-se um olhar clínico treinado, sendo uma das dificuldades que os enfermeiros enfrentam no dia a dia para atuar em situações de violência. Além disso, os autores também elencam outras realidades, como a de não expor ainda mais a criança, o adolescente e sua família ao agressor. A carência de formação/treinamento para toda a equipe multiprofissional e o excesso de trabalho que predispõe à síndrome de *burnout* são fatores que contribuem para que os casos de violência fiquem camuflados^(25)^.

No contexto dos serviços de saúde da rede pública, a porta de entrada para o SUS é a Estratégia Saúde da Família, e este espaço tem uma posição privilegiada para identificação de vítimas que sofrem maus-tratos. Desse modo, é de grande importância que esses profissionais sejam orientados como detectar os casos e como dar seguimento para que a vítima receba a assistência necessária^(15)^. Contudo, os laços firmados entre usuários e profissionais devem ser vistos e tratados como pontos positivos para percepção, monitoração e intervenção dos casos de violências às crianças e adolescentes^(26)^. Segundo a Fiocruz^(27)^, qualquer profissional que identifique uma vítima de violência necessita realizar a notificação junto ao SINAN e que nenhum caso pode ser subnotificado, considerando que se trata de uma notificação de caráter compulsório e que o seu preenchimento é uma dimensão do cuidado em saúde. Assim, a experiência do profissional de saúde é relevante para identificação dos casos, seja por meio da identificação das lesões ocasionadas na vítima ou por meio da conversa com a vítima e com os familiares^(27)^.

O Princípio do Melhor Interesse da Criança (PMICA) é uma ferramenta comumente utilizada em tomada de decisão que envolva crianças e/ou adolescentes, especialmente no âmbito jurídico, e mesmo que os participantes deste estudo sejam profissionais de saúde de nível superior, essa é uma temática pouco usual na prática clínica. É utilizado a nível mundial por profissionais que atuam com o público infanto-juvenil, aplicando-se no sistema de justiça^(28)^.

A dificuldade para a realização de notificação nos serviços de saúde da rede privada também é evidenciada. Os resultados do estudo realizado por Pires *et al.* ^(28)^, cujos participantes eram pediatras do Rio Grande do Sul que atuavam nas redes públicas e privadas, revelaram que alguns participantes da rede privada não efetuavam a notificação compulsória por motivos de pouco acesso a informações sobre a temática, envolvimento emocional, medo de envolvimento legal e ausência de suporte institucional eficaz no setor privado.

Diante do exposto, o objeto de estudo é o *Child Abuse and Neglect Reporting Self-Efficacy Questionnaire* (CANRSE), que mensura a autoeficácia dos profissionais de saúde para notificação de casos de abuso e negligência infantil. O CANRSE foi desenvolvido por Lee *et al.* em 2012^(29)^, Sydney, Austrália, com o objetivo de avaliar a autoeficácia para as notificações dos profissionais de enfermagem de casos suspeitos ou conhecidos de abuso e negligência contra crianças e adolescentes. O CANRSE, na sua versão original, é constituído por 55 itens e três dimensões: confiança para notificação dos casos suspeitos; confiança para notificação dos casos conhecidos, ambas com 20 itens; e confiança em que a notificação possa produzir resultados positivos para a criança e a família, com 15 itens^(30,31)^. Para o processo de adaptação do CANRSE, foram seguidas as etapas de tradução, síntese das traduções, realizadas pelos dois tradutores e um observador de gravação, retrotradução, comitê de revisão, pré-teste e submissão de todo o processo aos autores do instrumento^(32-34)^.

Este estudo está alinhado aos Objetivos do Desenvolvimento Sustentável, em seu objetivo 16, que tem como proposta “Promover sociedades pacíficas e inclusivas para o desenvolvimento sustentável, proporcionar o acesso à justiça para todos e construir instituições eficazes, responsáveis e inclusivas em todos os níveis”^(35)^.

## OBJETIVOS

Adaptar culturalmente o CANRSE para o Brasil.

## MÉTODOS

### Aspectos éticos

Esta pesquisa está inserida no projeto de pesquisa intitulado “Autoeficácia para a Notificação de Abuso e Negligência Infantil”, aprovado por um Comitê de Ética em Pesquisa Envolvendo Seres Humanos.

### Desenho, período e local do estudo

Estudo metodológico de aplicação clínica^(36,37)^, desenvolvido entre 2023 e 2024 nas cinco regiões do Brasil.

### População ou amostra; critérios de inclusão

Constitui-se na população deste estudo profissionais de saúde com nível superior de formação e que trabalham ou já trabalharam por, no mínimo, um ano no atendimento de crianças e adolescentes no Brasil. Para composição da amostra, os participantes foram distribuídos em 14 subconjuntos, formados segundo categorização dos profissionais de saúde em dois subconjuntos, tais como entre um ano ou menos do que quatro anos de formação e aqueles com cinco ou mais anos de formação, com cada um desses subconjuntos subdivididos em sete agrupamentos dos itens do CANRSE, a saber: 1 a 5; 6 a 10; 11 a 15; 16 a 20; 21 a 25; 26 a 30; 31 a 35.

Para cada um dos subgrupos formados, foram considerados no mínimo três participantes, com amostra estimada de 42 para cada uma das cinco regiões do Brasil, resultando em um valor estimado de 210 profissionais de saúde.

### Tradução e retrotradução para o português do Brasil

O processo de tradução e retrotradução dos itens do CANRSE seguiu o proposto por Guillemin *et al.* ^(33)^ e a adaptação proposta por Ferrer *et al.* ^(34)^. Para a tradução, foram considerados dois pesquisadores brasileiros com conhecimento da temática e com domínio do idioma inglês. O comitê de especialistas foi composto por cinco pesquisadores brasileiros com título mínimo de doutorado, experiência em tradução, validação de instrumentos, domínio na temática em questão, com conhecimento dos objetivos da pesquisa e também com domínio do idioma inglês. Na retrotradução, atuou um profissional de tradução juramentada, devidamente registrado na Junta Comercial do Estado de São Paulo.

### Validação de face da versão traduzida para o Brasil

Nesta etapa, foi utilizado o método de avaliação semântica proposto pelo grupo DISABKIDS^(38)^, adaptado para o Brasil pelo Grupo de Pesquisa sobre Medidas em Saúde do Conselho Nacional de Desenvolvimento Científico e Tecnológico (GPEMSA-CNPq)^(39)^. Os participantes do estudo realizaram a avaliação do CANRSE por meio da folha de impressão geral disponibilizada pelo DISABKIDS^(38)^, adaptada para o Brasil pelo GPEMSA-CNPq^(39)^, contendo três questões subjetivas e quatro questões objetivas. Houve também respostas em relação à folha de impressão específica, em que os participantes de cada agrupamento responderam às três questões (Tabela 1). Em relação à clareza e pertinência ou representatividade e relevância, as respostas foram descritas, segundo escala tipo Likert, em quatro opções, sendo 1 o pior julgamento do questionário, e 4, o melhor. Foram consideradas satisfatórias ou muito satisfatórias em relação à clareza e pertinência/representatividade do questionário respostas 3 ou 4, respectivamente.

**Tabela 1:** Distribuição dos participantes em relação às questões constantes na folha específica da validação semântica segundo itens do CANRSE-35 BR e categoria de maior tempo de atuação profissional junto ao público infanto-juvenil, Brasil, 2023

Itens do *Child Abuse and Neglect Reporting Self-Efficacy Questionnaire*-35BRASIL	Isto é importante para a sua situação?				O (A) senhor (a) teve dificuldade para entender essa questão?		O (A) senhor (a) poderia me dizer, em suas palavras, o que essa questão significa?
Subconjunto 1 (n =18)		Sim	Às vezes	Não	Sim	Não	
1		15	1	0	3	14	
2		14	2	1	2	15	
3		14	2	0	4	12	
4		14	3	0	4	13	
5		15	2	0	5	12	
Subconjunto 2 (n =14)							
6		12	1	0	3	10	
7		12	1	0	2	10	
8		12	0	0	7	5	
9		11	0	0	2	9	
10		10	0	0	3	7	
Subconjunto 3 (n =10)							
11		8	0	0	1	8	Fiquei em dúvida se a questão quer dizer que em casos de ter uma suspeita de abuso físico eu posso contar com a opinião do meu coordenador ou se tenho convicção que é um direito meu quando buscá-lo mediante essa situação e contar com seu auxílio.
12		9	0	0	3	6	1. Em dúvidas de como notificar os casos de abuso sexual, posso pedir a opinião da minha supervisora. 2. Tive dúvida se a questão quer dizer que, em casos de ter um abuso sexual, eu posso contar com a opinião do meu coordenador ou se tenho convicção que é um direito meu quando buscá-lo mediante essa situação e contar com seu auxílio.
13		7	1	0	2	7	
14		8	1	0	4	5	
15		9	0	0	4	5	
Subconjunto 4 (n = 8)							
16		8	0	0	3	5	
17		8	0	0	2	6	
18		8	0	0	2	6	
19		7	1	0	2	6	
20		8	0	0	2	6	
Subconjunto 5 (n =11)							
21		7	2	2	1	8	Não consegui compreender a respeito do princípio do melhor interesse da criança.
22		8	1	2	2	8	
23		7	2	2	2	7	
24		7	2	2	2	7	Princípio do melhor interesse da criança está confuso.
25		8	0	3	2	7	Princípio do melhor interesse da criança - confuso.
Subconjunto 6 (n = 8)							
26		7	0	0	3	4	A questão é confusa, talvez porque o assunto de melhor interesse da criança seja um assunto pouco falado pelos profissionais de saúde.
27		7	0	0	2	5	
28		6	0	0	2	4	
29		6	0	0	2	4	
30		6	0	0	2	4	
Subconjunto 7 (n = 6)							
31		5	1	0	2	4	
32		5	1	0	0	6	
33		5	1	0	0	6	
34		5	1	0	0	6	
35		5	1	0	0	6	

### Procedimento para coleta de dados

Nesta fase, para a formação do comitê de juízes, foram consultados *websites* das Instituições de Ensino Superior do país, e os profissionais foram selecionados por meio das informações constantes na Plataforma *Lattes.* Foram selecionados participantes das regiões Nordeste, Sul e Sudeste (São Paulo e Minas Gerais). Cada participante recebeu uma carta convite via *e-mail* com esclarecimentos sobre a pesquisa e sobre o dia da reunião de consenso. Para a coleta de dados da etapa de avaliação semântica, os participantes receberam uma carta convite por meio do *e-mail* e por meio das redes abertas explicando a respeito da pesquisa. O consentimento de participação foi via formulário eletrônico junto à plataforma *Research Electronic Data Capture* (REDCap), com a escolha da opção “sim” após leitura do Termo de Consentimento Livre e Esclarecido (TCLE), e só após isso o participante teve acesso ao instrumento disponibilizado na plataforma. Cada uma das etapas foi avaliada pelos participantes do grupo de pesquisa GPEMSA (Figura 1).

**Figura 1:** Fluxograma sobre as etapas para a disponibilização da coleta de dados por meio no REDCap, Ribeirão Preto, São Paulo, Brasil, 2023

## RESULTADOS

Em relação à tradução e retrotradução, a pesquisadora responsável pelo desenvolvimento do instrumento concordou com o conteúdo de cada item, preservando o construto mensurado. No entanto, fez as sugestões de substituir “denunciar” por “notificar” e de unificar as dimensões “confiança para notificação dos casos suspeitos” e “confiança para notificação dos casos conhecidos”, tornando o CANRSE, na versão brasileira, constituído por duas dimensões com 35 itens. A versão do CANRSE para o Brasil foi denominada CANRSE-35BR (Figura 2).

**Figura 2:** Modelo Teórico a ser confirmado segundo análise fatorial da versão adaptada para o Brasil do *Child Abuse and Neglect Reporting Self-Efficacy Questionnaire* (CANRSE-35BR)

No total, o estudo contou com 217 acessos, das cinco regiões do Brasil, ao instrumento disponibilizado na plataforma REDCap, porém 75 (34,6%) finalizaram as respostas à folha de validação específica. Na fase da avaliação geral, 126 profissionais de saúde responderam às 35 questões do CANRSE-35BR, sendo a região Nordeste a que obteve o maior número de participantes: 78 (61,9%).

Sobre o nível de formação dos participantes, a maioria tinha pós-graduação (101; 80,2%), e quanto à formação profissional, a maioria (67; 53,2%) era profissional de enfermagem. Entre os participantes, 56 (44,4%) possuíam entre um e quatro anos de maior tempo de atuação com o público infanto-juvenil; e 70 (55,6%) possuíam maior tempo de atuação superior a cinco anos.

O CANRSE-35BR foi muito bem avaliado pelos participantes considerando o questionário bom ou muito bom (111; 88,1%), com questões fáceis de entender (93; 73,8%), sem nenhuma dificuldade com as opções das respostas (94; 74,6%) e muito importantes para a situação (108; 85,7%). Também, a maioria (106; 84,1%) não gostaria de mudar algo; não quis acrescentar alguma coisa (115; 91,3%) e quis responder a todas as questões (123; 97,6%) do questionário.

Em relação às questões discursivas, os resultados são apresentados a seguir:

Item: “O (A) senhor (a) gostaria de mudar alguma coisa no questionário?”. Entre os participantes, 18 (14,3%) relataram querer mudar algo no questionário e sugeriram um esclarecimento sobre o “melhor interesse da criança e da família”; Item: “O (A) senhor (a) gostaria de acrescentar alguma coisa no questionário?”. Algumas poucas sugestões de acréscimos foram realizadas por dez (7,9%) dos participantes, com a criação de um campo para que o profissional possa se expressar mais e a inclusão das perguntas:

O (A) senhor (a) tem certeza do acompanhamento psicossocial das famílias das crianças/adolescentes suspeitas ou confirmadas de violência infantil?

O (A) senhor (a) tem certeza que pode notificar casos suspeitos ou confirmados de violência infantil por meio do disque 100, mantendo seu nome em anonimato?

O (A) senhor (a) tem segurança em identificar os abusos e negligência?

Considerando que identificar é tão importante como notificar abuso e negligência infantil, um dos participantes sugeriu acrescentar as perguntas:

O (A) senhor (a) tem vivências prévias de notificação?

Houve apreensão de conhecimento a respeito ainda na graduação?

Tais sugestões foram discutidas e, a decisão foi por não as acatar, visto alterarem o conteúdo do instrumento e serem sugeridas por uma minoria dos participantes.

A finalidade da pergunta foi identificar se o profissional tem desde a sua formação acadêmica envolvimento nesta temática, aplicando atualmente enquanto profissional formado. Item: “Teve alguma questão que o (a) senhor (a) não quis responder?”. Neste item, houve duas respostas de participantes que não quiseram responder às questões. Porém, os mesmos não especificaram quais as questões, tão pouco o motivo, e como as perguntas não tinham obrigatoriedade de respostas, eles continuaram a pesquisa até a avaliação semântica.

Participaram de toda a avaliação semântica 75 (34,6%) profissionais que, seguindo o método DISABKIDS adaptado para o Brasil^(39)^, inicialmente responderam às dez questões sociodemográficas. Após, responderam 35 itens do CANRSE-35BR, seis perguntas da folha de validação geral e toda a folha de validação específica.

A distribuição dos participantes desta fase, em relação ao maior nível de formação, incluiu 17 (22,7%) com graduação e 58 (77,3%) com pós-graduação (especialização, residência, mestrado, doutorado e pós-doutorado). Em relação ao gênero, 55 (73,3%) se declararam do sexo feminino; 14 (18,7%) se declararam do sexo masculino; e seis (8,0%) preferiram não declarar o gênero. Sobre o local de trabalho, a maioria (61; 81,3%) dos participantes atua profissionalmente na zona urbana; quatro (5,3%) trabalham na zona rural; nove (12,0%) trabalham nas duas localidades (zona rural e zona urbana); e um (1,3%) participante não informou o seu local de trabalho.

Por meio da folha de avaliação específica, os participantes avaliaram cada item do CANRSE-35BR sobre a importância dos itens para a sua realidade como profissional de saúde (Isto é importante para a sua situação?) a respeito da dificuldade para compreender o item “O (A) senhor (a) teve dificuldade para entender essa questão?”. Também foi oportunizado aos participantes expressar, com suas palavras, o que o item ao qual estava respondendo significava (O (A) senhor (a) poderia me dizer, em suas palavras, o que essa questão significa?) (Tabela 1).

Os resultados apresentados na Tabela 1 mostram que, para cada um dos itens, a maioria dos respondentes os julgou “importantes para a situação”, com o mínimo de respostas positivas para esta categoria iguais a 14 (77,8%), 10 (71,4%), 7 (70,0%), 7 (87,5%), 7 (63,6%), 6 (75,0%) e 5 (83,3%) para os subconjuntos de 1 a 7, respectivamente. Em relação às dúvidas, estas se associaram ao PMICA. Dos respondentes dos itens 11 e 12, quatro participantes expressaram que tiveram dúvidas no entendimento, pois não sabem se a questão trata sobre eles terem conhecimento de que podem contar com o apoio do gerente/supervisor para a realização da notificação em situações de abuso e/ou negligência ou se, quando há situações como estas, o gerente/supervisor trabalha em conjunto, ofertando apoio e subsídios para a notificação.

## DISCUSSÃO

A clareza e a objetividade de um instrumento de medida são fatores primordiais para considerá-lo bom. Textos longos e/ou frases extensas com muita riqueza de detalhes podem resultar em uma leitura monótona e exaustiva, causando dispersão do respondente e comprometimento em sua utilização^(40)^. Keszei *et al*.^(41)^ afirmam que a utilização de termos ambíguos e/ou vagos pode gerar respostas sem precisão e desapropriadas.

Neste trabalho, a unificação das dimensões “confiança para notificação dos casos suspeitos” e “confiança para notificação dos casos conhecidos” em “confiança para notificação dos casos suspeitos e conhecidos” reduziu em aproximadamente 36% o número inicial de 55 itens, tornando o CANRSE, na versão brasileira, um instrumento objetivo, constituído por duas dimensões, com 35 itens.

Em relação à relevância, clareza e compreensibilidade, o CANRSE-35BR foi muito bem avaliado pelos 126 participantes nesta fase, com 111 (88,1%) considerando-o bom ou muito bom, com questões fáceis de entender (93; 73,8%), sem qualquer dificuldade com as opções das respostas (94; 74,6%) e muito importantes para a situação (108; 85,7%). Ainda, 115 (91,3%) não quiseram acrescentar alguma coisa no questionário, e 123 (97,6%) quiseram responder a todas as questões.

Em relação às questões discursivas, os participantes tiveram a oportunidade de expressar suas opiniões a respeito do CANRSE-35BR, além de poder fazer sugestões e modificações no mesmo. Mais uma vez, os resultados foram muito satisfatórios, com poucas sugestões realizadas pelos participantes, ratificando a validade de face do instrumento.

Em relação à questão dissertativa da fase específica da avaliação semântica, os participantes do estudo tiveram a oportunidade de expressar com suas palavras o significado do item. O resultado relevante se associa ao PMICA, tema considerado complexo e de difícil entendimento por alguns participantes.

Embora 108 (85,7%) dos participantes tenham avaliados os demais como relevantes para a sua situação, os itens que abordaram o PMICA foram apontados como não importantes. A expressão “melhor interesse da criança/adolescente” é uma tradução do inglês “*The Best Interests of the Child”*, sendo utilizada pela primeira vez, em 1924, na Declaração dos Direitos da Criança de Genebra^(42)^. O PMICA apareceu originalmente no texto da Convenção Internacional das Nações Unidas sobre os Direitos da Criança, 1989, quando foram apresentadas as obrigações dos Estados para com a infância, determinando o mínimo que cada nação deveria garantir às suas crianças e adolescentes, em vigor a partir de 1990 (UNICEF)^(43)^.

O PMICA é importante quando a criança/adolescente se encontra exposto a situações de maus-tratos, negligências, vivenciando o processo de adoção, guarda ou abrigamento. Ações que sensibilizem os profissionais quanto à eficácia da notificação são essenciais, pois a notificação da violência contra crianças e adolescentes constitui um primeiro passo no caminho das ações de controle do agravo^(44,45)^.

É por meio da avaliação semântica, ou validade de face, que existe a possibilidade de realizar os ajustes no instrumento ao qual se pretende adaptar, possibilitando que ele se torne o máximo compreensível à população ao qual se destina. Nesta etapa, também podem ser identificadas dificuldades no entendimento em alguns termos, por se tratar de palavras que não são da vivência da população-alvo^(46,47)^. Os resultados apresentados mostraram que o processo de tradução para o português falado e escrito no Brasil e retrotradução do CANRSE preservou a equivalência conceitual dos itens com a versão adaptada, apresentando validade de face.

### Limitações do estudo

Neste estudo, apesar de o convite à participação ter sido estendido a todos os profissionais de saúde, a maioria dos participantes corresponde aos profissionais enfermeiros, com pouca participação dos demais. Ademais, compreendendo que a temática PMICA é mais trabalhada nas questões jurídicas, seria essa a justificativa para os profissionais de saúde não terem familiaridade com a temática “princípio do melhor interesse”.

### Contribuições para a área da enfermagem, saúde ou política pública

Nos resultados do estudo, foram apresentadas fragilidades relacionadas ao conhecimento do PMICA pelos profissionais de saúde participantes, o que sugere o fortalecimento de políticas públicas voltadas ao processo formativo e à educação continuada na perspectiva de elaboração de estratégias visando à notificação e monitoramento eficazes de casos de violência contra o público infantojuvenil.

Também, em uma perspectiva interprofissional e multidisciplinar, a disponibilização de um instrumento para mensuração da autoeficácia das notificações dos casos de abuso e de negligência infantil pode impactar positivamente diversas áreas, como a prática clínica na enfermagem, possibilitando tomada de decisão eficaz, subsidiando o gerenciamento dos casos de violência, a resposta das autoridades competentes e a segurança das crianças, adolescentes e seus familiares em vulnerabilidade, bem como dos profissionais que notificam.

## CONCLUSÕES

Este estudo finaliza o processo de adaptação cultural do CANRSE para o contexto estudado, um instrumento que, após ser validado em relação aos aspectos psicométricos, constituir-se-á em ferramenta válida e fidedigna que poderá ser utilizada para a mensuração da autoeficácia dos profissionais de saúde para notificação de casos suspeitos ou conhecidos de abuso e de negligência infantil, e para identificação de fatores associados ou para a avaliação de intervenções para seu aprimoramento.

A versão traduzida para o Brasil do CANRSE, denominada CANRSE-35 BR, apresentou validade de face e evidências de aplicabilidade, e pode ser considerada adaptada culturalmente para o país. Mais uma vez, o método de avaliação semântica, que oportuniza “dar voz” aos participantes do estudo, permitiu o alcance do objetivo do processo de adaptação cultural de instrumentos com rigor metodológico.

Apesar de não se constituir em objetivo do presente estudo, recomenda-se a necessidade de capacitações com vistas à detecção e notificação de casos suspeitos ou conhecidos de violência contra crianças e adolescentes dos demais tipos de violência. Para garantir uma melhor qualidade nos dados analisados, torna-se necessário realizar o monitoramento das notificações, contínua preparação dos profissionais de saúde, correta identificação de casos suspeitos e preenchimento adequado da ficha de notificação. Ações que sensibilizem os profissionais quanto à eficácia da notificação são essenciais, pois a notificação da violência contra crianças e adolescentes constitui um primeiro passo no caminho das ações de controle do agravo^(47)^.

Assim, simultaneamente à verificação da estrutura fatorial e validação do CANRSE, ressalta-se a necessidade de desenvolvimento de estudos que abordem sobre o PMICA no contexto dos atendimentos de serviços de saúde, visto ficar claro neste estudo ainda a falta de conhecimento de alguns dos participantes sobre este assunto.

## Data Availability

Os dados de pesquisa não estão disponíveis.
